# Speciation, Diversification, and Coexistence of Sessile Species That Compete for Space

**DOI:** 10.1371/journal.pone.0096665

**Published:** 2014-05-12

**Authors:** Namiko Mitarai, Els Heinsalu, Kim Sneppen

**Affiliations:** 1 Center for Models of Life, Niels Bohr Institute, University of Copenhagen, Copenhagen, Denmark; 2 Niels Bohr International Academy, Niels Bohr Institute, University of Copenhagen, Copenhagen, Denmark; 3 National Institute of Chemical Physics and Biophysics, Tallinn, Estonia; University of California-Irvine, United States of America

## Abstract

Speciation, diversification, and competition between species challenge the stability of complex ecosystems. Laboratory experiments often focus on one or two species competing under conditions where they may grow exponentially. Field studies, in contrast, emphasize multi-species communities characterized by many types of ecological interactions. A general problem is to understand conditions that support a dynamically maintained coexistence of many species in an ecosystem over a long time span. In the present paper we propose a lattice model of multiple competing and evolving sessile species. When allowing the interspecies interactions to mutate, we obtain coexistence of many species in a complex ecosystem, provided that there is a cost for each interaction. The diversity reached by the model incorporating speciation is found to be substantially higher than in the case when entirely new species appear due to immigration from outside of the considered ecosystem. The species self-organize their spatial distribution through competitive interactions to create many patches, implicitly protecting each other from competitively superior species, and speciation in each patch leads the system to high diversity. We also show that species that exist a long time tend to have a relatively small population, as this allows them to avoid encounter with competitive invaders.

## Introduction

Biological organisms cooperate and compete with each other forming complicated ecological systems with an intriguing ability to sustain themselves over long time-spans. In fact, this stability is not easy to understand as the interplay between exponential growth and competition produces an inherently unstable state. Accordingly, ecosystems consisting of more than a few species should tend to collapse into a low diversity state. In a seminal paper R. May pinpointed that this instability is weakened by reducing the number of interactions in the ecosystem [1]. One way to reduce interactions as well as exponential growth and competition is to introduce spatial segregation [2]–[9].

Many ecosystems consist of multiple interacting species that may form niches for each other [10]–[12], exemplified by the concept of keystone species [13], [13]–[15]. As a model for self-organized niche formation, a simplified description of competing lichen species on the two-dimensional surface of a rock has been introduced in Refs. [16], [17]. This model considers the spreading and competition of mutually exclusive species on a two-dimensional lattice. The competitive interactions between species are assigned randomly. Each lattice site contains a maximum of one species which attempts to colonize the neighboring sites. The invasion is possible only if the ecological interaction between the invader and the species that occupies the neighboring site allows it. In addition, new species are occasionally introduced at random positions, leading to a state of high diversity, provided that the likelihood for interactions is low [16], [17]. If the model is simulated in the well mixed situation by allowing interactions between spatially separated species, the high-diversity state collapses [16]; the space is essential for the high-diversity state. The short range interactions are one of the important sources for the high-diversity [7]. Another important factor is transient cyclic invasion that generates patches of isolated niches when it collapses [17].

In the present work, we investigate a two-dimensional evolution model for sessile species, where new species are not introduced from outside, but instead evolve from the already existing species. The major result observed for the model investigated in Refs. [16], [17] is found also now, i.e., the high diversity state is stable when the species invasive interactions are sufficiently inhibited. The model allows us to combine allopatric and sympatric speciation through a self-organized segregation of species into isolated patches. The mutations in segregated patches allow neutral evolution and lead to evolutionary divergence of the properties of the spatially separated species.

## Model

We investigate a model representing the evolution of an ecosystem that initially consists of 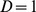 species only, occupying one randomly chosen lattice site of a 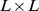 square lattice with periodic boundary conditions. For simplicity, we assume that a species is characterized by its ecological interactions, expressed by the interaction matrix 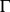 as explained below. Namely, the only phenotype we focus on, is the interspecies interaction; we ignore the difference between the genotype and phenotype. The differences in the ecological interactions do not necessary indicate the difference in, for example, interbreeding ability. Therefore the word *strains* instead of *species* may be sometimes more suitable when the difference between diverging lineages is small, though after long time the accumulation of divergent mutations tend to promote the origin of widely different species. In the following we use the term *species* only for simplicity.

At each update a site 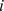 is selected randomly among the occupied sites. With probability 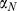 the species 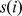 *mutates* to change its ecological interactions. If no mutation takes place, a site 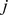 is selected randomly among the four nearest neighbors of site 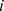. If the species 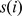 can invade the species 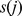, 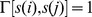, then the corresponding *invasion* takes place, i.e., site 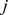 is updated by setting 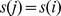. Here 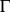 is a matrix that represents possible interactions between the species, which remain fixed once they are introduced. It is assumed that if 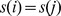 then 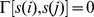 and that an empty site can always be invaded, 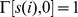. One Monte Carlo time step is defined as 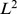 repetitions of the described updates.

In the case of mutation the interaction matrix elements of the new species 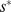 coming from the ancestor 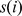 are assigned according to the following rules:

1. for any existing species 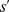 one initializes 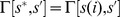 and 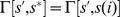;
2. one randomly selected element of 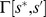 or 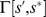 is set to 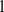, i.e., if the value was 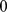 then now the mutant 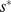 can invade a species 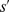 or can be invaded by a species 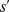 which had no interaction link with the ancestor 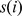, respectively; if the value was already 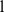 then nothing changes;
3. for each 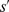 the elements 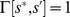 and 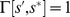 are set to 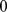 with probability 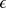, representing a cost in maintaining interactions;
4. finally, we set 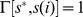 and 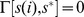, i.e., it is assumed that the mutant 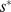 can invade its ancestor 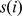 but the ancestor cannot invade its mutant.

In short, the new species 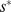 inherits most of the phenotypical features of its ascendant 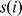, represented in the competition network, with small random modifications. This is in contrast to the previously studied model in Refs. [16], [17], where the interactions for a new invading species was assigned randomly with a pre-determined interaction probability 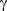.

The rule 4 that new species can always invade its ancestor while the ancestor cannot invade its mutant is motivated by the following: One could consider that one site is already a collection of many individuals. Then, 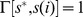, 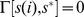 is the combination that the new species will succeed to fixate. If 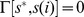, 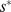 is unlikely to take over already existing 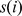 in the site. Even if 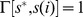, if 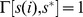 also holds then it is expected to be hard for the species 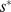 to win over majority species 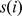. In addition, if we open for the stand-off relations, namely 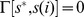 and 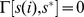, then this will create a lot of small static dusts of new species in the ancestor species region. Since the separated patches are crucial for higher species diversity as we have already seen in the original model [16], [17], having this additional possibility is expected to increase the diversity further more, but such an effect may be too artificial.

The time scale of mutation is expected to be much slower than the time scale of interspecies interactions in an ecosystem. Therefore, in some of our simulations we investigate the quasi-static limit, which effectively is equivalent to the limit 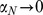. This approximation focuses on the evolution of a new species, whereas its effect on population redistribution is speeding up cyclic interactions to re-establish a representative frozen state [16] before the next mutation. New species appear through mutations only after all the activity of invasions has died out, because when multiple species compete for a finite region, the stochasticity of the dynamics eventually makes one of the species to take over. In most of the cases this process takes quite a short time, about 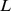 steps, which is the time scale for a front to sweep the whole system. However, there are occasional events where several species compete dynamically for the same area for a long time period, often due to a short cyclic relationship. To save the computation time, we shorten the long-lasting competition by temporally preventing one of the randomly chosen active species to invade any other species after typically 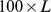 time steps. It should be noted that the one species state is an absorbing state in this limit; therefore we use the high-diversity state obtained by a finite 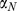 simulation as the initial condition for the quasi-static simulation [16], [17]. In order to reduce the computation time, the simulations with small values of 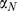, including the quasi-static simulations, were performed by the event-driven type algorithm, where the possible events are listed and time to the next event was drawn accordingly from the exponential distribution. We have verified that this method gives statistically the same results as the described random sequential updates. Typically the model is simulated for a long time before a reliable analysis of steady state properties can be made. The subsequent section analyzes aspects of this steady state dynamics.

## Results

### Time evolution of the system

**Figure 1:**
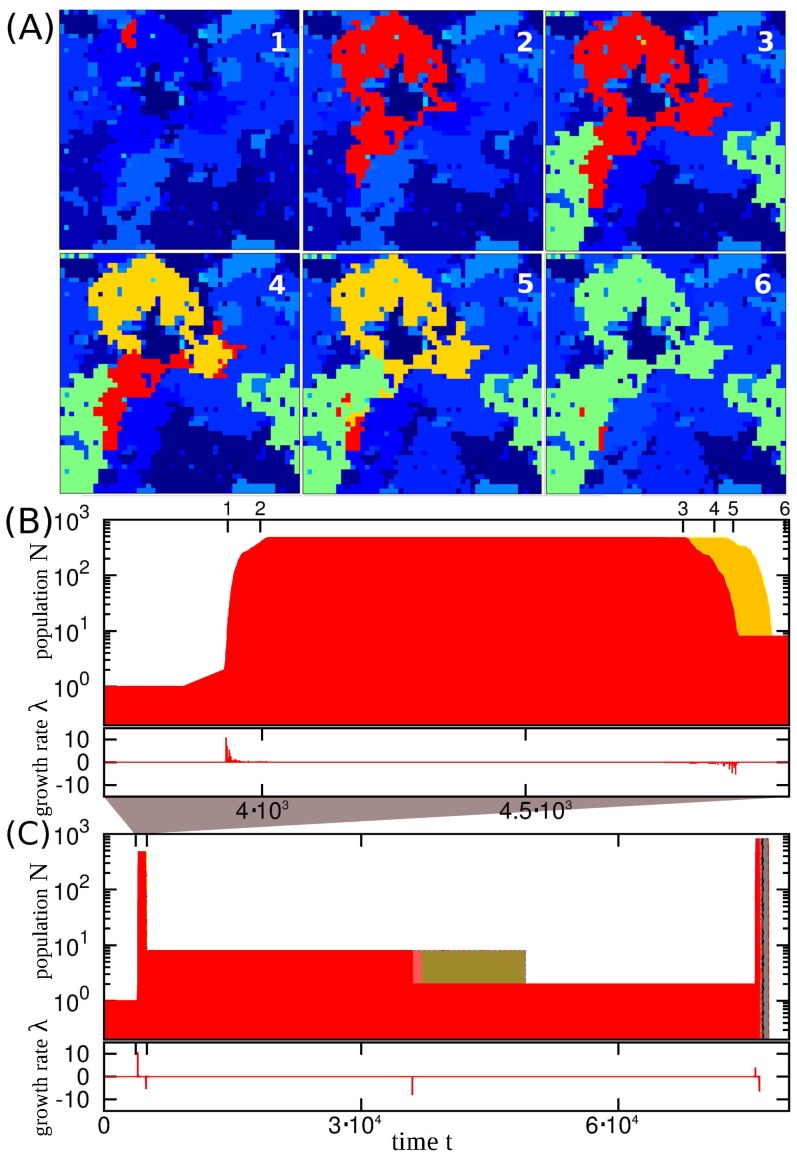
Example of the time evolution in the steady state. For a system with size 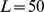, link penalty 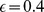 and mutation rate 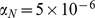. (A) Snapshots of the system at the times marked in top of panel B. (B) Time evolution of the population size, 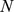, of the species marked in red in panel A. The population size, 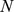, of its offspring species is shown in yellow. Below the panel is depicted the growth rate, 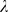, defined through Eq. (1). (C) Time evolution of the population size of the same species and its offspring species as in panel B on a longer time scale. There are many more offspring species, but ultimately the whole lineage goes extinct.

In order to understand the dynamics of the system, let us start by investigating the time evolution of a stochastic realization of the system. Figure 1A presents snapshots of the system at different times after the steady state has been reached. Figures 1B and 1C present the time evolution of population size 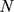 of the species marked in red in Fig. 1A and of its offspring species during a shorter and a longer time interval, respectively. The rest of the species in the snapshots are shown with various shade of blues, except for one of the off-springs (yellow) and the species that invade the yellow species (green).

By comparing the snapshots 1 and 2 in Fig. 1A, we can observe the expansion of the species marked in red in the territory that was initially occupied by one of the species marked in blue. This expansion corresponds to a step-like change in its population size, 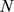, in Fig. 1B. Subsequently, a long silent co-existence of the two species takes place (compare snapshots 2 and 3 in Fig. 1A), where the population size, 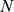, of the red species remains constant (Fig. 1B). The mutation rate 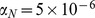 corresponds to one mutation per 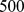 time-steps among about 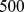 lattice sites of the red population. The change in population size coincides with mutations in red species with this time scale; there occurs a mutation of the red species with a speciation into a yellow descendant (see snapshot 3 in Fig. 1A). The yellow species does not expand into the territory of the blue species, but it invades its ancestor region (compare snapshots 3 and 4 in Fig. 1A). However, the expansion of the green species separates the red species and its yellow offspring (see snapshot 5 in Fig. 1A) at time 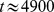. Finally, the green species brings the yellow one into extinction, letting at the same time the red species to survive (see snapshot 6 in Fig. 1A).

Figure 1C illustrates the fate of the red species on a longer time-scale. The dynamics is persistently punctuated [18], with small time intervals of rapid changes followed by long periods where the population size, 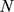, is constant. In terms of the fitness concept, the growth rate 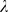 of the population,
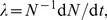
(1)is associated to the fitness 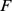 as 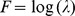, and 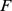 deviates from 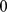 in an erratic way only for short time intervals. The inserts below the population size, 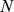, in Figs. 1C and 1B show the growth rate, 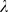, of the population, which can be negative when the species is exposed to its competitively superior species. Whereas the measurement of 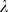 in a short time scale naturally reflects the contemporary environment, the assignment of the fitness in a long time scale is meaningless. Instead, the survival of a species in a longer time scale depends on the extent to which the species hedges its option by occupying separate patches.

**Figure 2:**
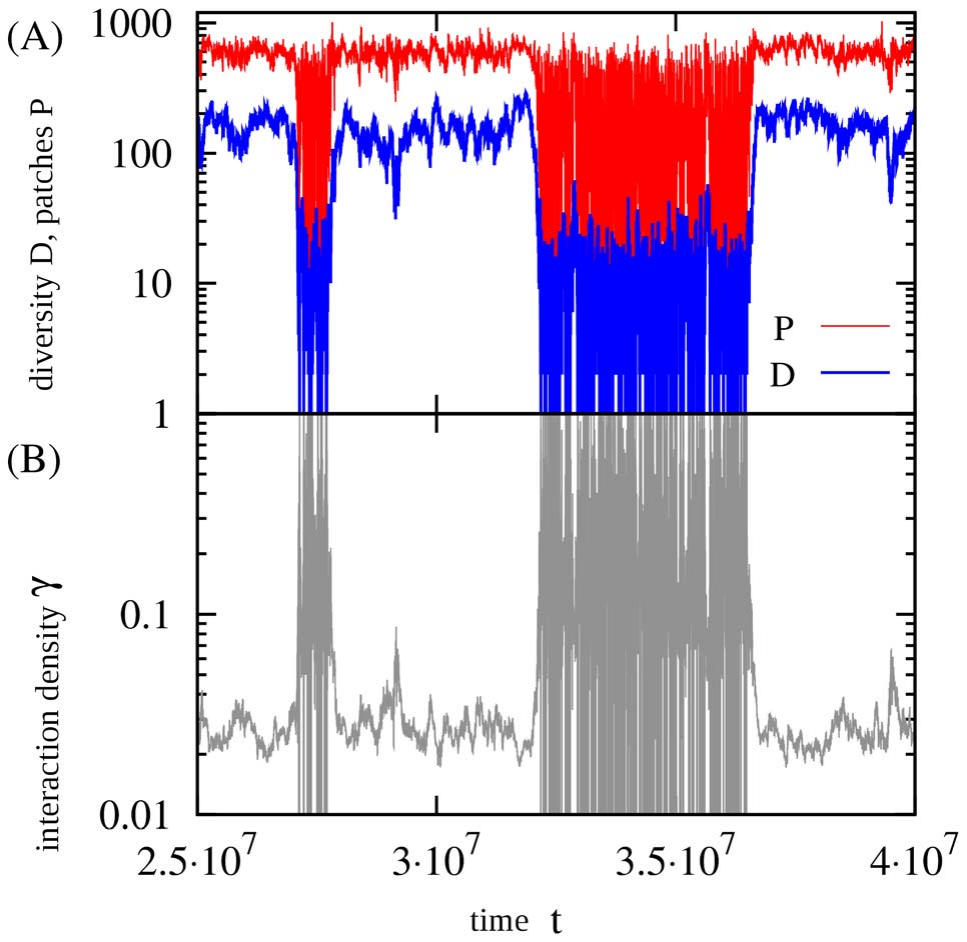
Time evolution in the steady state. (A) Diversity (number of species), 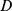, and number of patches, 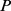, and (B) interaction density 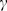. 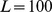, 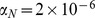, and 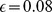.

Figure 2 examines the larger scale dynamics of the system with size 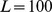 for a value of 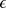 leading to the situation when the system may persist at both low and high diversity. Figure 2A shows the time dependence of the diversity, 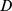, and the number of patches, 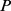, in the system after the steady state has been reached. One can observe the switching between a state with low diversity and highly varying patchiness, and a state with high diversity and high patchiness. This may resemble the bi-stability found earlier in the model of Refs. [16], [17] for the interaction density 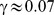 (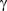 is the probability that an element in 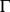-matrix takes the value 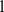), but in the present case 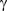 changes with time (see below). There can also be a finite size effect, regarding that the previous model required 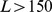 to have a stable high diversity state [16], [17].

At any time, the system may be characterized by the number of species, 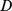, and by the interaction density, 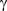, that can be calculated from the contemporary interaction matrix 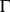 at that time point as follows: 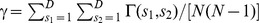 (remember that the diagonal element of the interaction matrix, 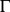, is zero). Figure 2B examines 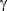, which takes the value 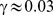 in the high diversity state and is oscillating between 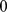 and 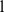 in the low diversity state. Figure 2B therefore illustrates a positive feedback between the high diversity and the difficulty in maintaining potential interactions with many species. For the high diversity state, the value of 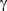 can be estimated quite accurately (data not shown) from the steady state condition by adding and removing links assuming that the matrix is represented by 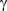:
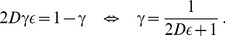
(2)


### Steady state properties

**Figure 3:**
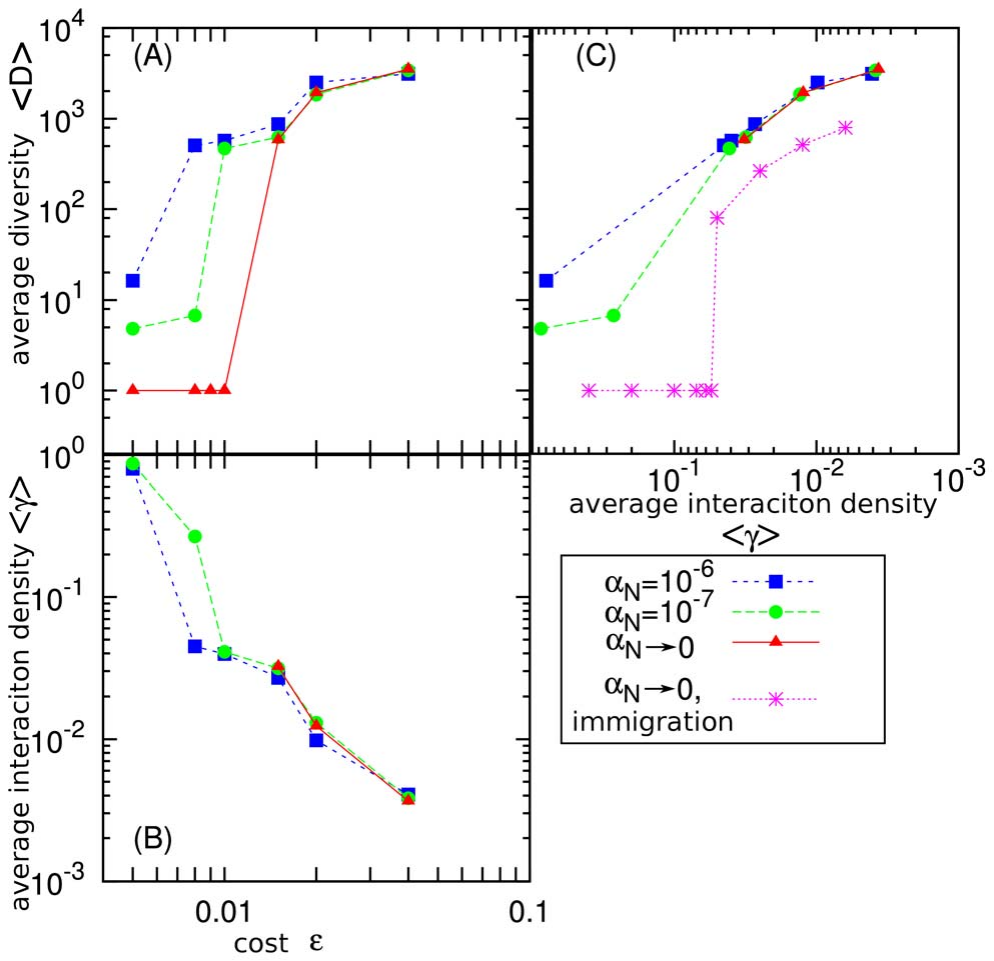
The long-time average of the systems behavior. (A) Diversity, 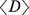 and (B) interaction density, 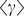 are shown as a function of 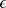 for 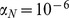, 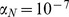, and in the quasi-static simulation for 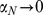 (the system size is 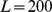). (C) The same data as in panels A and B plotted as 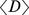 versus the measured interaction density 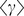: Note that 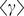 decreases to the right. In the case of 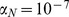 and in the quasi-static simulations we started from the high diversity state obtained for 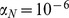 and 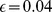 and simulated the relaxation to a constant value. For comparison, the results for the model without heredity, studied in Ref. [16], are also plotted in the quasi-static limit. For a smaller system size, e.g., 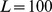, the high diversity states shifts toward 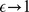 as 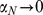, indicating that the high diversity state is not stable in the quasi-static limit for a too small system (data not shown).

The steady state properties of the system are presented in Fig. 3 for a lattice size 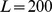; all panels in Fig. 3 examine three different values of the mutation rate 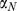, in order to investigate the limit 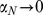. To see the transition between the low and high diversity states, we study in Figs. 3A and 3B, respectively, the average diversity, 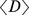, and the average interaction density, 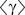, versus 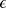 (the averages are taken over a long time after the steady state has been reached). As one can see from Fig. 3A, in the quasi-static limit, 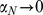, there is a sharp transition from low to high diversity state between the values 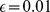 and 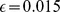. The figure indicates also that higher speciation rates, 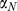, are associated to the high diversity state that can be maintained at substantially lower interaction penalties 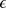. It was confirmed that the well-mixed version of the model did not give a stable high-diversity state, namely the space is necessary (data not shown).

The comparison of Figs. 3A and 3B shows that all high diversity states are associated to 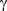 values below a critical value that is about 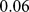, apparent also from the function 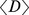 versus 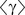 in Fig. 3C. For comparison, Fig. 3C shows also the behavior of 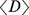 versus 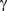 in the non-evolutionary model of Ref. [16], recapitulating the critical 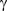 value for this version of the model. In general, the qualitative first order transition between the low and high diversity states is found to be robust against the rule that species interaction is “inherited”, i.e., copied to new species with only small changes. The main quantitative difference with the non-evolutionary model of Ref. [16] is that the present version of the model leads to approximately three times larger value of 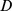 in the high diversity state (see Fig. 3C).

In order to gain more understanding about the system, it is useful to investigate also the Hamming distance between the species 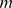 and 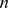, characterizing their similarity,
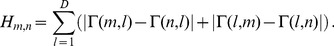
(3)


The Hamming distance in the high diversity state is very close to the expected value for randomly assigned 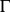-matrix with the interaction density 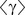,
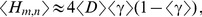
(4)when averaged over the neighboring species (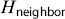) and over all pairs of species in the system (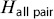), as shown in Table 1. This result indicates that species mutate many times neutrally before spreading substantially in space. This local accumulation of mutations suppresses spatial correlations between the phenotypes of neighboring species.

**Table 1:** Hamming distance in the high diversity state in quasi-static 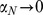 case.

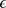	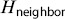	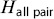	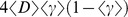
0.015	70.7	70.2	73.3
0.02	95.2	92.0	93.9
0.04	49.9	49.7	50.7

### Following lineages

**Figure 4:**
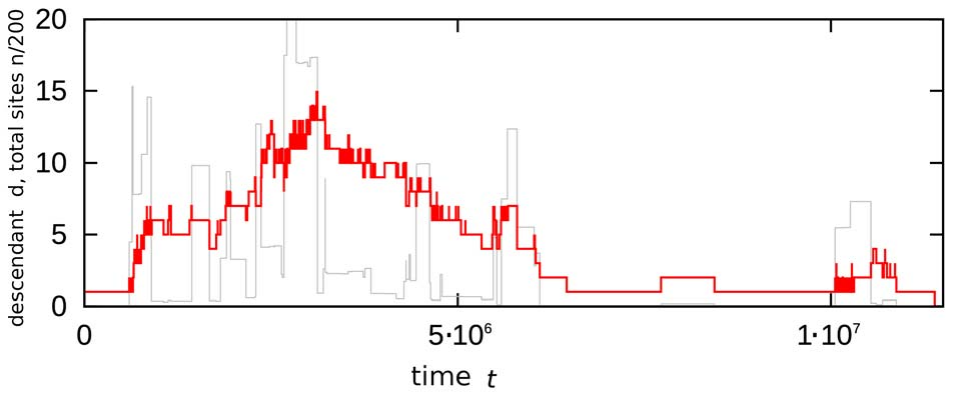
Time evolution of the lineage of a species that starts with one successful species at 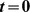. Number of descendant species, 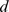, (red thick line) and total number of occupied sites, 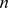, (scaled down by factor 200, grey thin line) versus time. The parameters are: 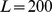, 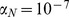, and 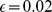.

The proposed evolution model allows one to follow descendants of individual species and therefore to examine to what extent the change in population size is correlated with the diversity and the survival of a lineage. Figure 4 shows one case history, following a fairly successful species that for a limited time period has more than ten different offspring species occupying up to 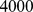 lattice sites on a 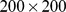 lattice. Whereas the number of offspring species develops quite gradually, one can notice that the population changes are intermittent. Remarkably, the lineage mostly persists in a stage with relatively few individuals.

**Figure 5:**
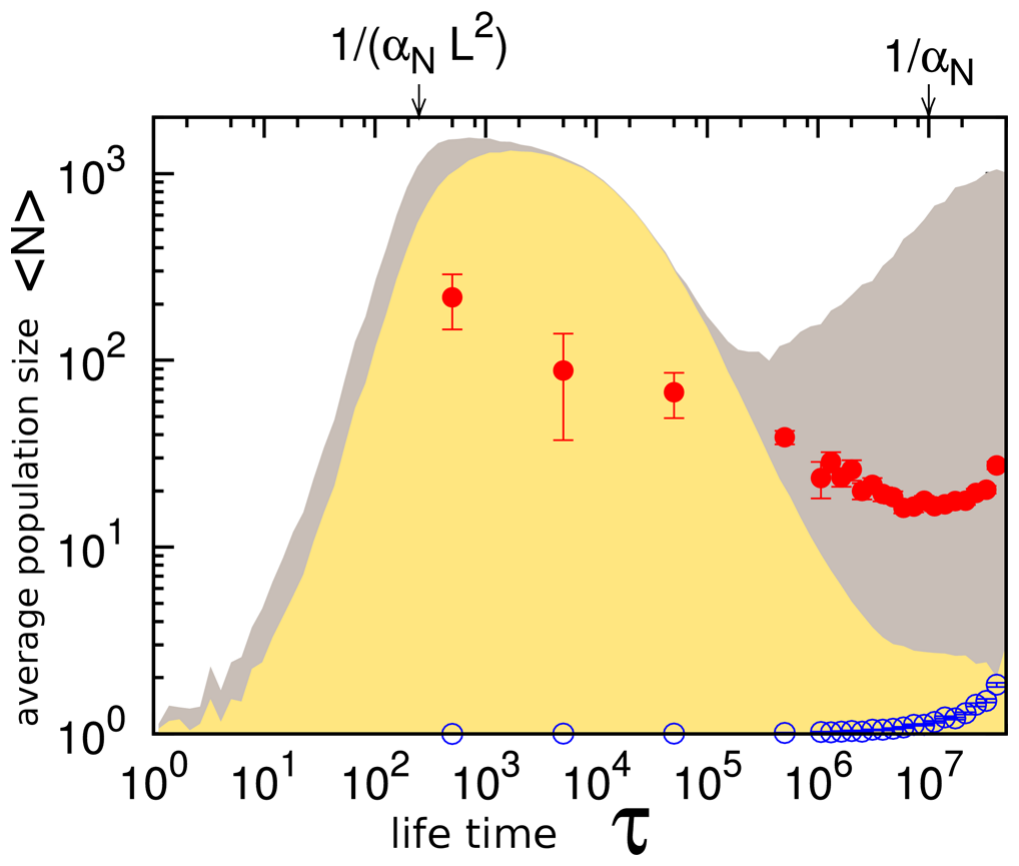
Time average of the population size. 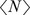
 is as a function of the life time, 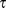, of a species (yellow) and as a function of the life time of all its descendants (red circles) in a system with 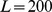, 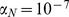, and 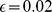. The grey shadow indicates the maximum value of the population size reached by the species with the given life time. For 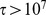 each site in the system has experienced at least one mutation event, whereas the first mutation in the system occurs at 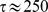. The open blue circles show the time averaged number of descendants, a number that grow beyond one for 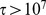.

Figure 5 systematizes the population statistics of species and their families as a function of their life-time (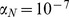, 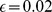, and 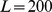). Interestingly, the species that exist only for a rather short time (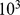
–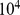 time units) are those whose population sizes are the largest. In contrast, the species that exist for sufficiently long time to allow speciation (blue circles) mostly have small population sizes. We emphasize that also the maximum population of a species is declining when its total existence time becomes long (data not shown). Instead, the red circles depict the average population size, 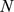, of the whole lineage starting with a single species. Again, one can observe a decline in population as survival time is longer, a pattern that is also consistent with the case story plotted in Fig. 4.

## Discussion

In the present work we investigated a model of competition between sessile species that evolve. As we have defined a species in terms of interaction options then the evolution is represented by small changes (mutations) in these links. The focus of the proposed model is speciation, where a population of a species is spatially disconnected by interspecific competition, and this disjunction facilitates subsequent divergence by allowing each patch to mutate and develop differently. This speciation scenario resembles to some extent the allopatric speciation for evolution of plants [19], [20]. However, here it is determined by other species, and not by mountain rages or other non-biological factors. As far as we know, this is a new proposal of a mechanism to create long-term isolated populations (meta-populations). The closest ecological data related to spatially driven speciation is the studies on how the dynamical change of geological environment affects the speciation events [21]–[23]. These studies consider the situation where the geological changes are due to external causes such as climate change, but it will be interesting to include the possibility that the change of species spatial distribution is caused by the interspecies interaction.

We have observed that the population in each patch evolves in a series of replacements where new mutants replace the old ones. Most of these replacements are phenotypically neutral in the given environment of the patch, allowing large scale evolutionary meandering in analogy to neutral evolution [24]–[26]. As a consequence of this extensive neutral development within each path, the species in neighbor patches are typically as different from each other as any random pair of species. When the inhabitant of a patch finally becomes capable to invade a neighbor, it emerges as a species that is entirely different from the species that originally established the patch. Therefore the model behaves to a large extent as the immigration model investigated in Ref. [16], where a new completely randomized species was introduced at each “mutation step”. However, the present evolution model has substantially higher diversity in the high diversity state, provided that the parameters are chosen so that in the two models the interaction density 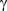 has the same value. The higher diversity in the evolution model is accompanied by many more small (order of size one) patches than the immigration model.

Finally, we would like to underline that the model implicitly incorporates an effective “fitness” with unusual properties. First of all, the fitness is clearly context dependent as the growth and the existence of a species entirely relies on the surrounding species. In this sense the model bears resemblance to the model of Ref. [27], where the stability of a species was defined in terms of its neighbors. Secondly, the exponential growth of a population in the current model is limited to a very short period, whereas a frozen steady state dominates most of the evolutionary trajectory of any species. We believe that our focus on short bursts and collapses captures the large scale evolution more accurately than a fitness defined by a potential for exponential growth.

The presented evolution scenario also suggests a reinterpretation of the Red Queen hypothesis of Van-Valen, who proposed that an observed constant species survival with geological time reflects a survival race where everybody has to improve just to maintain an unchanged survival chance [28]. We have observed that the survival of species lineages through time does not depend on obtaining large populations or on proliferation on a short time-scale; instead, it depends on the extent to which a species hedges against hostile attack by splitting its population into isolated patches. Small populations of isolated species are often predicted to exist for very long time intervals. This is in contrast to the common observation that larger population indicates longer survival [29]. Of course, in reality, a species with too small population will not be stable; in the present model, one site is already assumed to be enough to sustain the species as long as there is no other species attacking it, indicating that one site is already above the minimum population size to avoid purely random extinction due to fluctuation of the population size. If the present mechanism of species survival is in effect, there can be a non-monotonic dependence of the species long-time survival on its population.

Although we have addressed the problem of non-motile species it is tempting to speculate about its overall behavior in the context of the fossil record of all animal species. It has been found in Ref. [30] that the long time survival of genera does not correlate with their short time “success”. Although these data deal with duration of taxonomic orders with a short time “success” quantified by genera diversity, they also suggest a conceptual separation of the short time success from the long time survival.
